# Trimester-specific phthalate concentrations and glucose levels among women from a fertility clinic

**DOI:** 10.1186/s12940-018-0399-5

**Published:** 2018-06-14

**Authors:** Tamarra M. James-Todd, Yu-Han Chiu, Carmen Messerlian, Lidia Mínguez-Alarcón, Jennifer B. Ford, Myra Keller, John Petrozza, Paige L. Williams, Xiaoyun Ye, Antonia M. Calafat, Russ Hauser, EARTH Study Team

**Affiliations:** 1 Department of Environmental Health, Harvard T.H. Chan School of Public Health, 665 Huntington Ave., Bldg. 1, 14th Floor, Boston, MA 02115 USA; 2 Department of Epidemiology, Harvard T.H. Chan School of Public Health, Boston, MA 02115 USA; 3 Division of Women’s Health, Department of Medicine, Connors Center for Women’s Health and Gender Biology, Brigham and Women’s Hospital and Harvard Medical School, Boston, MA 02120 USA; 4 Department of Nutrition, Harvard T.H. Chan School of Public Health, Boston, MA 02115 USA; 5 Fertility Center, Department of Obstetrics, Massachusetts General Hospital, Boston, MA 02125 USA; 6 Department of Biostatistics, Harvard T.H. Chan School of Public Health, Boston, MA 02115 USA; 7 Division of Laboratory Sciences, Centers for Disease Control and Prevention, Atlanta, GA 30341 USA

**Keywords:** Phthalates, Blood glucose levels, Pregnancy, Infertility, Endocrine disruptors

## Abstract

**Background:**

Subfertile women are at increased risk of glucose intolerance in pregnancy. Based on epidemiologic studies, exposure to certain phthalates is associated with diabetes, elevated glucose, and increased insulin resistance.

**Objectives:**

To evaluate the association between urinary phthalate metabolites and pregnancy glucose levels in women seeking medically assisted reproduction.

**Methods:**

We evaluated 245 women participating in a prospective cohort study based at a large fertility clinic who delivered live births and had data on pregnancy urinary phthalate metabolite concentrations and blood glucose levels. Urinary phthalate metabolite concentrations were from single spot urine samples collected in 1st and 2nd trimesters. Blood glucose data was abstracted from medical records for non-fasting 50-g glucose challenge tests at 24–28 weeks gestation. Multivariable linear regression models were used to evaluate associations between 7 urinary phthalate metabolites in quartiles and mean glucose adjusted for potential confounders.

**Results:**

Eighteen percent of women had glucose levels ≥ 140 mg/dL. Second trimester monoethyl phthalate (MEP) concentrations were positively associated with glucose levels, with adjusted mean (95%CI) glucose levels of 121 mg/dl (114, 128) vs. 109 mg/dL (103, 116) for women in highest and lowest quartiles, respectively. Women in the highest quartile of second trimester mono-isobutyl phthalate (MiBP) concentrations had a mean glucose level 14 mg/dL lower compared to women in the lowest quartile. No other urinary phthalate metabolites were associated with glucose levels.

**Conclusions:**

MEP and MiBP—metabolites of diethyl phthalate and dibutyl phthalate, respectively—were associated with higher pregnancy glucose in subfertile women—a population at high risk of glucose intolerance in pregnancy.

**Electronic supplementary material:**

The online version of this article (10.1186/s12940-018-0399-5) contains supplementary material, which is available to authorized users.

## Background

Gestational diabetes mellitus (GDM) complicates ~ 7% of pregnancies in the United States [1]. Women with underlying causes of infertility such as polycystic ovarian syndrome (PCOS) and those conceiving with medically assisted reproduction represent a high risk group, with an increased risk of not only GDM, but also elevated glucose levels in pregnancy [2, 3]. Specifically, women conceiving with assisted reproductive technology have a risk of GDM that is up to 2 times higher than women conceiving naturally [4, 5]. Given that infertility affects more than 10–15% of all couples [6], the increased risk of GDM among a subfertile population remains a concern.

A growing body of literature suggests that any form of pregnancy hyperglycemia could lead to adverse outcomes, even without overt GDM [7, 8]. Women with elevated glucose levels, even without meeting the clinical criteria for GDM, are at increased risk of a variety of adverse health outcomes, including caesarean section, preeclampsia, and preterm birth [8, 9]. While lifestyle factors are considered important determinants of pregnancy hyperglycemia [10], some studies suggest that environmental factors such as endocrine disrupting chemicals (EDCs) might also be involved in hyperglycemia and diabetes [11–15]. Phthalates are a class of chemicals that may be associated with higher glucose levels [12].

Phthalates are used as plasticizers in the production of a variety of consumer products; low molecular weight phthalates are mainly used in cosmetics and personal care products, and high molecular weight phthalates in food packaging [16, 17]. Phthalates are also used in vinyl flooring, raincoats, car parts, and furniture [16, 17]. As such, phthalates are ubiquitous with over 90% of the U.S., European, and Canadian populations having detectable urinary concentrations of phthalate biomarkers [18–22].

Phthalates affect glucose metabolism through their ability to bind to human peroxisome proliferator activated receptors (PPAR) alpha and gamma, which could lead to upregulation of target genes associated with increased adipogenesis and insulin resistance with subsequent elevations in glucose levels [23]. Phthalates also modulate steroidogenesis [24, 25], as well as increase oxidative stress [26, 27], which are associated with increased GDM risk [28–30]. Three recent studies have evaluated the association between urinary phthalate metabolite concentrations and GDM and its related factors [31–33]. While two of these studies found no association between urinary phthalate metabolite concentrations and GDM [31, 32], the other study found an association only for second trimester mono-ethyl phthalate (MEP) concentrations and impaired glucose tolerance and excessive gestational weight gain—both risk factors of GDM [33]. None of these studies evaluated this research question among women at high risk of impaired glucose tolerance due infertility.

The purpose of this study was to prospectively evaluate the association between first and second trimester urinary phthalate metabolite concentrations and pregnancy glucose levels among women seeking medically assisted reproduction at a fertility clinic. We further assessed whether age, body mass index, or type of infertility treatment modified associations within this cohort.

## Methods

### Study population

The present study is a sub-analysis within the Environment and Reproductive Health (EARTH) Study, an ongoing, prospective study that recruited participants who sought infertility evaluation or treatment from the Massachusetts General Hospital Fertility Center. Women 18 to 46 years of age at enrollment were eligible to participate in the study. Among those referred to the research nurses by physicians and clinic staff, 60% agreed to participate and were enrolled into the study. Further details of the EARTH study are described elsewhere [34]. For this analysis, women were included if they had a singleton or twin pregnancy between 2005 and 2015, provided at least one urine sample for the measurement of phthalate metabolites during early and/or mid-pregnancy, and had electronic medical record data on a 50-g glucose challenge test (GCT). Of these, we excluded one woman who had a history of diabetes at baseline. We included 245 pregnant women who prospectively provided a total of 417 urine samples. Women who did not meet inclusion criteria (*n* = 166) had similar baseline characteristics as those included for the analysis.

### Ascertainment of clinical data

Clinical information was abstracted from the patient’s electronic medical record by trained study staff. The participant’s date of birth was collected at entry, and weight and height were measured by trained study staff. Body mass index (BMI) was calculated as weight (in kilograms) per height (in meters) squared. Women from the study achieved pregnancies by in vitro fertilization (IVF), intrauterine insemination (IUI), or naturally without medical intervention.

Every woman receiving obstetric care at the study hospital underwent GDM screening with a non-fasting, 50-g GCT at ~ 24 to 28 weeks of gestation (median: 27 weeks gestation). Women with blood glucose levels ≥140 mg/dL after GCT were considered to have impaired glucose tolerance (IGT) in accordance with Carpenter-Coustan criteria [35], and were recommended for further screening.

### Quantification of urinary phthalate metabolites

Spot urine samples were collected in each trimester using sterile polypropylene cups (median 1st trimester: 7 weeks and median 2nd trimester: 21 weeks). For the present analysis, we only used urine samples collected prior to or at the time of the GCT test. Specific gravity (SG) was measured using a handheld refractometer (National Instrument Company, Inc., Baltimore, MD, USA) to correct phthalate concentrations for urine dilution.

The urine was divided into aliquots, frozen at − 20 °C, and stored at − 80 °C. Samples were shipped on dry ice overnight to the CDC (Atlanta, GA, USA) for the quantification of concentrations of mono(2-ethylhexyl) phthalate (MEHP), mono(2-ethyl-5-hydroxyhexyl) phthalate (MEHHP), mono(2-ethyl-5-oxohexyl) phthalate (MEOHP), mono(2-ethyl-5-carboxypentyl) phthalate (MECPP), mono(3-carboypropyl) phthalate (MCPP), monocarboxyisooctyl phthalate (MCOP), monocarboxyisononyl phthalate (MCNP), monobenzyl phthalate (MBzP), monoethyl phthalate (MEP), mono-isobutyl phthalate (MiBP) and mono-n-butyl phthalate (MBP) using solid phase extraction coupled with high performance liquid chromatography-isotope dilution tandem mass spectrometry. The standard QA/QC procedures were previously described [36]. We calculated the molar sum of di(2-ethylhexyl) phthalate (DEHP) metabolites (∑DEHP) by dividing each metabolite concentration by its molecular weight and summing: [(MEHP*(1/278.34)) + (MEHHP*(1/294.34)) + (MEOHP*(1/292.33)) + (MECPP*(1/308.33))].

### Statistical analysis

Demographic characteristics of the study participants were reported using mean ± standard deviation (SD) or N (percentages). Urinary phthalate metabolite concentrations below the limit of detection (LOD) were replaced with a value equal to the LOD divided by square root of 2 prior to SG adjustment [37].

To adjust for urinary dilution, the following formula was used: Pc = P[(1.015–1)/(SG - 1)], where Pc is the SG-corrected phthalate metabolite concentration (μg/L), P is the measured phthalate metabolite concentration (μg/L), and 1.015 is the mean SG level in the study population. The glucose levels were log-transformed to meet the normality assumption of linear regression, and results were back transformed to improve interpretability. We categorized exposure in quartiles. To assess trimester-specific urinary phthalate metabolite concentrations based on potentially critical windows of exposure with respect to glucose tolerance, we assessed the associations with SG-adjusted urinary phthalate metabolite concentrations in quartiles at the first and the second trimesters separately.

We fit multivariable linear models to evaluate the association between quartiles of urinary phthalate metabolite concentrations and pregnancy glucose levels. Population marginal means were utilized to present the population average for each quartile adjusted for covariates in the model at their mean levels for the covariates [38]. Tests for trend were conducted across quartiles using the median of log SG-adjusted phthalate metabolite concentrations in each quartile as a continuous variable in the regression models.

Confounding was evaluated using directed acyclic graphs based on prior knowledge of potential confounding variables. The following covariates were considered for inclusion in the final model: age at GCT (years), pre-pregnancy overweight or obese (≥ 25 kg/m^2^ versus < 25 kg/m^2^), total physical activity (hr/week), race/ethnicity (non-white versus white), family history of diabetes (yes versus no), infertility diagnosis (female factor, male factor versus unexplained), and number of fetuses (2 versus 1) in a pregnancy. We also assessed effect modification by age at rapid fertility decline (< 37 years versus ≥ 37 years) [39]. In addition, BMI (< 25 kg/m^2^ versus ≥25 kg/m^2^) and treatment modes (IVF, IUI, versus natural conception) were assessed as potential effect modifiers. For these analyses, we used cross-product terms in the multivariable models.

We conducted several sensitivity analyses. First, we excluded 14 women without prospectively collected urine samples. In addition, we excluded 85 women with only one urine sample collected. In this subset of 159 women with prospectively collected urine samples with urinary phthalate metabolite concentrations measured at both 1st and 2nd trimesters, we evaluated the associations between trimester-specific urinary phthalate metabolites and glucose levels from the GCT. As an additional sensitivity analysis, we calculated the geometric mean of urinary phthalate metabolite concentrations across 1st and 2nd trimesters in this same subset of 159 women to assess average pregnancy phthalate metabolite associations with pregnancy glucose levels. Due to small numbers of women in our overall study population meeting the criteria for GDM based on Carpenter-Coustan criteria (*n* = 10 women) [35], we did not perform the analysis for GDM based on clinical diagnosis. However, we ran an additional sensitivity analysis using multivariable logistic regression evaluating urinary phthalate metabolite concentrations and an indicator of impaired glucose tolerance (GCT values ≥ 140 mg/dL; *n* = 44 women). The cut point of ≥ 140 mg/dL is the clinically-relevant threshold for additional screening for GDM based on the Carpenter-Coustan method [35]. In addition, we conducted a sensitivity analysis by restricting to singleton births, as well as excluding those taking metformin (*n* = 8) and those with a physician diagnosis of PCOS (*n* = 21). We also made additional adjustments for year of urine sample collection to account for potential changes in phthalate exposure throughout the years of the study.

In addition, to assess the possibility of confounding by dietary factors, we conducted a sensitivity analysis among a subgroup of women who had completed a validated 131-item food frequency questionnaire [40]. We adjusted for two dietary patterns score (i.e. Western and Prudent dietary patterns), total caloric intake and total fat intake [41]. All statistical analyses were conducted using SAS version 9.4 (SAS Institute Inc., Cary, NC). Two-sided *P* values < 0.05 were considered significant.

## Results

A total of 245 women, representing ~ 60% of the overall EARTH Study population, had data on urinary phthalate metabolites in the first (median: 7 weeks gestation) or second trimester (median: 21 weeks gestation), as well as glucose information from the 50-g GCT conducted as a part of the GDM screening test (median: 27 weeks gestation). The median difference in time between urine collection and GCT was 6 weeks. Overall, the average age of women was 35.3 years (SD: 3.8), with 30% of the population being overweight/obese (Table 1). The study population was predominantly white (87%) and college educated (88%). Approximately 57% of women underwent IVF during the current pregnancy, with 19% being pregnant with twins. Almost 20% of women had glucose levels ≥ 140 mg/dL from the 50-g glucose challenge tests, warranting additional GDM screening. Compared to women with glucose levels < 140 mg/dL, a greater proportion of women with glucose levels ≥ 140 mg/dL had an infertility diagnosis that was male factor (*p* = 0.01). In addition, a greater proportion of overweight/obese women (40%) had glucose levels ≥ 140 mg/dL. More Asian women had glucose levels ≥ 140 mg/dL (41% of Asian women compared to 16% of white women). However, BMI and race/ethnicity did not significantly differ by GCT categories.

**Table 1:** Baseline characteristics among 245 pregnant women in the Environment and Reproductive Health (EARTH) Study by second trimester glucose challenge test (GCT) level

Characteristic, N (%)	Total	GCT < 140 mg/dL	GCT ≥ 140 mg/dL	P value^1^
N	245	200	45	
Age at pregnancy (years)				0.59
Mean ± SD	35.3 ± 3.8	35.2 ± 3.8	35.6 ± 3.7	
Range	26.0–47.0	26.0–47.0	29.0–42.0	
Pre-pregnancy BMI (kg/m^2^)				
BMI < 25	171 (69.8)	144 (72.0)	27 (60.0)	0.15
BMI ≥ 25	74 (30.2)	56 (28.0)	18 (40.0)	
Smoking status				0.25
Never smoked	181 (73.9)	151 (75.5)	30 (66.7)	
Current smoker	58 (23.7)	45 (22.5)	13 (28.9)	
Former smoker	6 (2.5)	4 (2.0)	2 (4.4)	
Race				0.09
Caucasian	213 (86.9)	177 (88.5)	36 (80.0)	
Black/African American	5 (2.0)	4 (2.0)	1 (2.2)	
Asian	17 (6.9)	10 (5.0)	7 (15.6)	
Other	10 (4.1)	9 (4.5)	1 (2.2)	
Education				0.60
High school graduate or less	23 (9.4)	18 (9.0)	5 (11.1)	
Some college	7 (2.9)	5 (2.5)	2 (4.4)	
College graduate or higher	215 (87.8)	177 (88.5)	38 (84.4)	
Family history of diabetes	30 (12.2)	8 (17.8)	22 (11.0)	0.21
Infertility diagnosis				0.01
Male factor	68 (27.8)	48 (24.0)	20 (44.4)	
Female factor	78 (31.8)	64 (32.0)	14 (31.1)	
Decreased ovarian reserve	16 (6.5)	12 (6.0)	4 (8.9)	
Ovulatory	33 (13.5)	29 (14.5)	4 (8.9)	
Endometriosis	12 (4.9)	11 (5.5)	1 (2.2)	
Uterine	5 (2.0)	4 (2.0)	1 (2.2)	
Tubal	12 (4.9)	8 (4.0)	4 (8.9)	
Unexplained	99 (40.4)	88 (44.0)	11 (24.4)	
Treatment				0.81
IVF	138 (56.3)	111 (55.5)	27 (60.0)	
IUI	54 (22.0)	44 (22.0)	10 (22.2)	
Natural	53 (21.6)	45 (22.5)	8 (17.8)	
Fetus number of the pregnancy				0.54
One fetus	198 (80.8)	163 (81.5)	35 (77.8)	
Two fetuses	47 (19.2)	37 (18.5)	10 (22.2)	

Abbreviations: *GCT* 50-g glucose challenging test (non-fasting), *BMI* body mass index, *DOR* diminished ovarian reserve, *IVF* in vitro fertilization, *IUI* intrauterine insemination, *SD* standard deviation

^1^Kruskal-Wallis analyses (for continuous variables) and Fisher**’**s exact tests (for categorical variables) were used to test for associations by impaired glucose tolerance status.

In Table 2, we present the distribution of SG-adjusted trimester-specific urinary phthalate metabolite concentrations for 1st and 2nd trimesters for 7 metabolites and the ∑DEHP metabolites. A total of 208 women had urinary phthalate metabolite concentrations measured in the 1st trimester, while 209 had concentrations for the 2nd trimester. Overall, we found that median 2nd trimester concentrations were 38% higher for MEP compared to 1st trimester concentrations. MCPP, MCOP, and ∑DEHP concentrations were about 20–50% lower in 2nd trimester compared to first trimester. All other phthalate metabolite concentrations were similar for 1st and 2nd trimesters. Compared to a representative sample of U.S. females, concentrations of MEP and MCOP were higher in our study population, while most other concentrations were similar to those found in U.S. females [42]. Detection ranged from 92.7 to 100% for phthalate metabolites.

**Table 2:** Distribution of SG-adjusted trimester specific urinary phthalate metabolites measured during pregnancy among 245 women^1^ from EARTH study

Phthalate metabolites	Trimester	Number	Detection frequency^2^	SG-adjusted concentrations						
				geometric (SD)	min	25th	50th	75th	95th	max
MEP (ng/mL)	1	208	100%	43.6 (4.4)	3.33	15.3	31.96	96.54	586.74	12,600
	2	209	100%	60.2 (6.1)	2.96	21.99	47.99	144.2	1024	4791
MBP (ng/mL)	1	208	99.5%	10.9 (0.6)	<LOD	6.61	10.47	16.21	40.37	2186
	2	209	97.6%	11.8 (0.9)	<LOD	6.83	11.95	21	64.52	1699
MiBP (ng/mL)	1	208	96.6%	5.7 (0.3)	<LOD	3.45	5.82	9.97	19.6	52.85
	2	209	97.6%	5.7 (0.4)	<LOD	3.29	6.16	10.11	24.36	152.35
MBzP (ng/mL)	1	208	95.7%	3.0 (0.2)	<LOD	1.51	2.87	5.02	18.2	285.06
	2	209	91.9%	2.9 (0.2)	<LOD	1.41	2.8	5.37	26.21	369.6
MCPP (ng/mL)	1	208	98.1%	4.9 (0.5)	<LOD	1.67	3.81	12.52	77.78	651.72
	2	209	96.7%	3.7 (0.3)	<LOD	1.69	2.9	6.46	45.13	486.5
MCOP (ng/mL)	1	192	97.9%	28.2 (2.9)	<LOD	10.46	28.28	76.39	322	687.27
	2	190	97.4%	21.9 (2.3)	<LOD	7.7	20.38	60.39	322	659.17
MCNP (ng/mL)	1	192	93.2%	4.2 (0.3)	<LOD	1.87	3.36	7.66	29.65	239
	2	190	92.6%	3.5 (0.3)	<LOD	1.75	2.99	6.3	30.05	209.07
∑DEHP(nmol/mL)	1	208		0.2 (0.02)	0.02	0.09	0.15	0.36	2.71	17.89
	2	209		0.1 (0.01)	0.01	0.07	0.12	0.23	1.5	8.21

Abbreviations: *min* minimum, *max* maximum, *SG* specific gravity, *N* number of women = number of urine samples, *MEP* monoethyl phthalate, *MBP* mono-n-butyl phthalate, *MiBP* mono-isobutyl phthalate, *MBzP* monobenzyl phthalate, *MCPP* mono-3(carboxypropyl) phthalate, *MCOP* monocarboxyisooctyl phthalate, *MCNP* monocarboxyisononyl phthalate, *DEHP* di-2(ethylhexyl) phthalate

^1^Of 245 women, 208 women provided 1st trimester urine sample, and 209 women provided 2nd trimester urine sample.

^2^Percent of phthalate metabolite concentrations above the limits of detection (LODs). LODs were 0.4–0.8 μg/L for MEP; 0.4–0.6 μg/L for MBP; 0.2–0.3 μg/L for MiBP; 0.2–0.3 μg/L for MBzP; 0.1–0.2 μg/L for MCPP; 0.2–0.7 μg/L for MCOP; 0.2–0.6 μg/L for MCNP; 0.5–1.2 μg/L for MEHP, 0.2–0.7 μg/L for MEHHP and MEOHP; and 0.2–0.6 μg/L for MECPP.

**Fig. 1:**
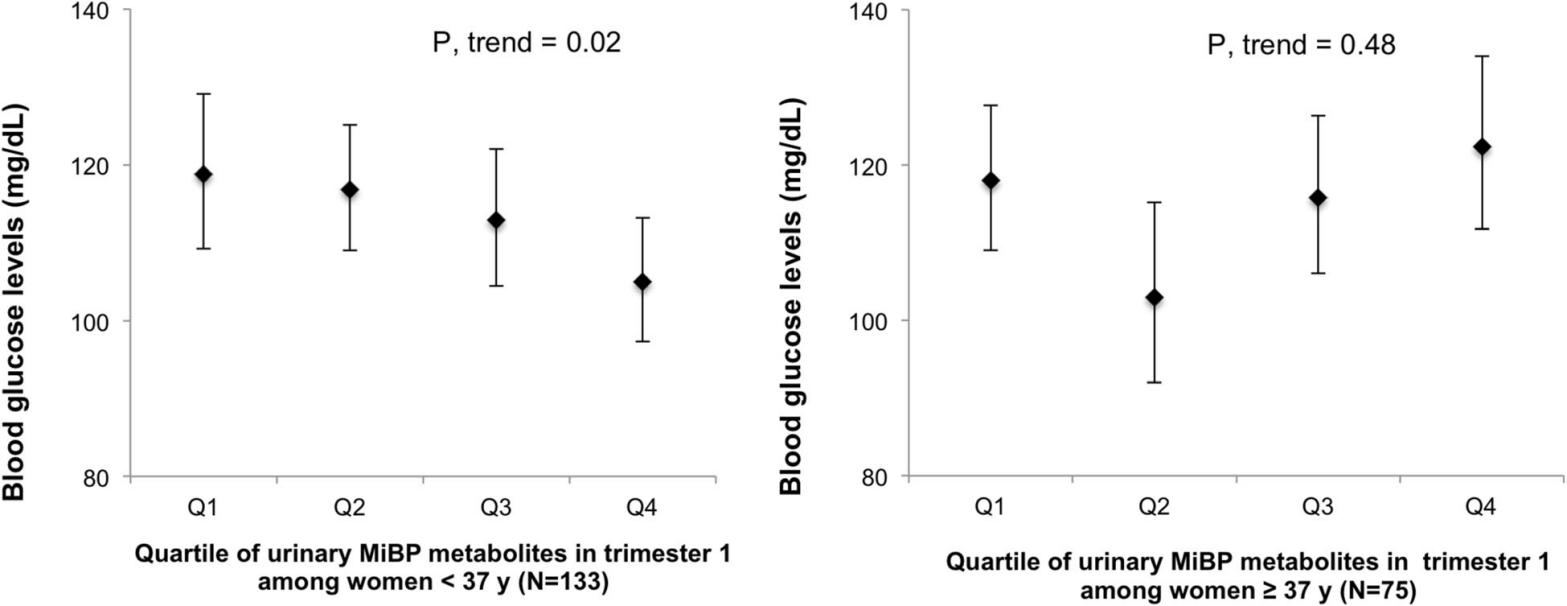
Age-stratified mean blood glucose levels across quartiles of urinary MiBP concentrations measured during the 1st trimester. Legend: Abbreviations: MiBP, mono-isobutyl phthalate. Models were adjusted for maternal age (years), overweight/obese (yes, no), total physical activity (hr/week), race (white, non-white), family history of diabetes (yes, no), infertility diagnosis (male factor, female factor, unexplained) and number of fetus (1,2)

**Fig. 2:**
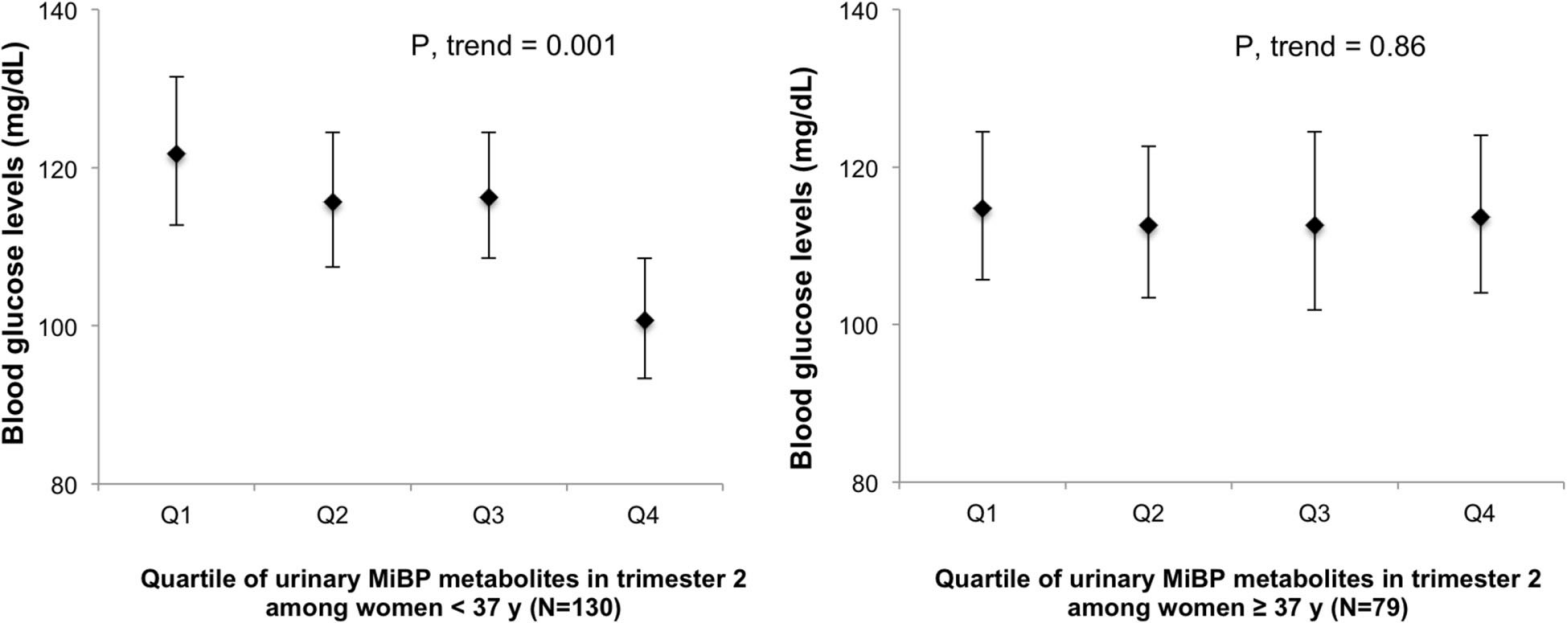
Age-stratified mean blood glucose levels across quartiles of urinary MiBP concentrations measured during 2nd trimester. Legend: Abbreviations: MiBP, mono-isobutyl phthalate. Models were adjusted for maternal age (years), overweight/obese (yes, no), total physical activity (hr/week), race (white, non-white), family history of diabetes (yes, no), infertility diagnosis (male factor, female factor, unexplained) and number of fetus (1,2)

When evaluating the association between 1st trimester urinary phthalate metabolite concentrations and blood glucose levels from the late 2nd trimester 50-g GCT, we did not observe any associations between urinary phthalate metabolites and glucose levels (Table 3). However, we found 2nd trimester MEP concentrations to be positively associated with glucose levels (Table 3). For example, women with MEP concentrations in the highest quartile had average glucose levels that were 12 mg/dL higher compared to women in the lowest quartile after adjusting for age, BMI, total physical activity, race/ethnicity, infertility diagnosis, family history of diabetes, and number of fetuses (adj. Glucose for Q4 of MEP: 121 mg/dL; (95% CI: 114, 128) v. adj. Glucose for Q1 of MEP: 109; (95% CI: 103, 116); p for trend: 0.02). MiBP was inversely associated with glucose levels, with women who had highest concentrations having average glucose levels that were 14 mg/dL lower compared to women with the lowest concentrations of MiBP (adj. Glucose for Q4 of MiBP: 105 mg/dL; (95% CI: 99, 111) v. adj. Glucose for Q1 of MiBP: 119; (95% CI: 113, 126); p for trend: 0.003). None of the other urinary phthalate metabolites showed associations with glucose levels from the GCT (Table 3). In stratified analyses, we found effect modification by maternal age assessed as an indicator of rapidly declining fertility based on < 37 years versus ≥ 37 years for the association between MiBP and pregnancy glucose levels (see Figs. 1 and 2). Specifically, younger women had significantly lower glucose levels compared with older women, with associations being driven by women in the highest quartile of MiBP (p for interaction: 0.04). However, associations were not modified by BMI and IVF status (data not shown).

**Table 3:** Adjusted mean blood glucose levels across quartiles of urinary phthalate metabolite concentrations measured during the 1st trimester and 2nd trimesters

1st trimester SG-adjusted phthalate metabolites (range)	Population means of blood glucose in mg/dL (95%CI) across quartiles of phthalate metabolites measured at the 1st trimester (*n* = 208)		2nd trimester SG-adjusted phthalate metabolites (range)	Population means of blood glucose in mg/dL (95%CI) across quartiles of phthalate metabolites measured at the 2nd trimester (*n* = 209)	
	Unadjusted	Adjusted ^1^		Unadjusted	Adjusted ^1^
MEP (μg/L)			MEP (μg/L)		
Q1 (3.6, 16.3)	114 (107, 121)	114 (108, 121)	Q1 (3.2, 23.1)	108 (102, 115)	109 (103, 116)
Q2 (16.5, 34.2)	113 (106, 120)	115 (108, 122)	Q2 (23.6, 50)	112 (105, 119)	113 (107, 120)
Q3 (34.3, 103)	110 (103, 117)	109 (103, 116)	Q3 (51.4, 155)	112 (105, 119)	111 (105, 117)
Q4 (104, 13,500)	120 (113, 128)	118 (111, 125)	Q4 (156, 5133)	123 (116, 130)*	121 (114, 128)
P-trend^2^	0.19	0.55	P-trend^2^	0.003	0.02
MBP (μg/L)			MBP (μg/L)		
Q1 (<LOD, 7.1)	115 (108, 122)	116 (109, 123)	Q1 (<LOD, 7.2)	111 (105, 118)	112 (106, 119)
Q2 (7.1, 11.2)	113 (106, 120)	112 (105, 118)	Q2 (7.3, 12.7)	111 (105, 119)	113 (106, 120)
Q3 (11.2, 17.3)	117 (109, 124)	116 (109, 123)	Q3 (12.8, 22.4)	115 (108, 122)	114 (107, 121)
Q4 (17.4, 2342)	113 (106, 120)	113 (107, 120)	Q4 (22.5, 1820)	116 (110, 124)	115 (109, 122)
P-trend^2^	0.80	0.72	P-trend^2^	0.26	0.50
MiBP (μg/L)			MiBP (μg/L)		
Q1 (<LOD, 3.7)	118 (111, 125)	118 (111, 125)	Q1 (<LOD, 3.4)	118 (111, 126)	119 (113, 126)
Q2 (3.7, 6.2)	112 (106, 120)	114 (107, 121)	Q2 (3.5, 6.6)	115 (108, 122)	115 (109, 122)
Q3 (6.2, 10.6)	114 (108, 122)	114 (107, 121)	Q3 (6.6, 10.8)	114 (107, 121)	115 (109, 122)
Q4 (10.7, 56.6)	112 (105, 119)	111 (104, 117)	Q4 (10.9, 163)	107 (100, 113)*	105 (99, 111)
P-trend^2^	0.36	0.15	P-trend^2^	0.02	0.003
MBzP (μg/L)			MBzP (μg/L)		
Q1 (<LOD, 1.6)	113 (106, 121)	113 (107, 120)	Q1 (<LOD, 1.5)	110 (104, 117)	111 (105, 118)
Q2 (1.6, 3.1)	115 (108, 123)	117 (110, 124)	Q2 (1.5, 3.0)	116 (109, 123)	117 (110, 124)
Q3 (3.1, 5.4)	110 (103, 117)	110 (103, 116)	Q3 (3.0, 5.7)	110 (103, 117)	110 (104, 117)
Q4 (5.4, 305)	118 (111, 125)	117 (110, 124)	Q4 (5.9, 396)	118 (111, 125)	116 (109, 123)
P-trend^2^	0.60	0.82	P-trend^2^	0.26	0.56
MCPP (μg/L)			MCPP (μg/L)		
Q1 (<LOD, 1.8)	114 (107, 121)	115 (108, 122)	Q1 (<LOD, 1.7)	110 (104, 117)	112 (106, 119)
Q2 (1.8, 4)	117 (110, 125)	115 (109, 122)	Q2 (1.8, 3.1)	114 (107, 121)	113 (107, 120)
Q3 (4.2, 13.2)	117 (110, 125)	118 (111, 125)	Q3 (3.1, 6.9)	115 (109, 123)	116 (109, 122)
Q4 (13.7, 698)	109 (102, 116)	109 (102, 115)	Q4 (7.0, 521)	114 (107, 121)	113 (107, 120)
P-trend^2^	0.24	0.19	P-trend^2^	0.52	0.84
MCOP (μg/L)^3^			MCOP (μg/L)^3^		
Q1 (<LOD, 11.1)	116 (108, 124)	115 (108, 123)	Q1 (<LOD, 8)	112 (105, 120)	113 (106, 120)
Q2 (11.3, 29.8)	115 (108, 123)	115 (108, 122)	Q2 (8.2, 21.8)	109 (103, 117)	111 (105, 118)
Q3 (30.7, 79.8)	113 (106, 121)	113 (106, 121)	Q3 (21.9, 64.7)	114 (107, 122)	113 (106, 120)
Q4 (83.9, 736)	109 (102, 116)	109 (102, 116)	Q4 (66.5, 706)	113 (106, 121)	113 (106, 120)
P-trend^2^	0.17	0.19	P-trend^2^	0.65	0.93
MCNP (μg/L)^3^			MCNP (μg/L)^3^		
Q1 (<LOD, 2)	110 (103, 117)	110 (103, 117)	Q1 (<LOD, 1.9)	112 (105, 119)	115 (108, 122)
Q2 (2, 3.6)	120 (112, 128)	119 (112, 127)	Q2 (1.9, 3.2)	112 (105, 119)	109 (102, 116)
Q3 (3.6, 8.2)	112 (105, 120)	113 (106, 120)	Q3 (3.2, 6.7)	114 (106, 121)	115 (108, 123)
Q4 (8.2, 256)	111 (104, 118)	111 (104, 118)	Q4 (6.7, 224)	112 (105, 120)	110 (104, 117)
P-trend^2^	0.75	0.83	P-trend^2^	0.86	0.59
∑DEHP			∑DEHP		
Q1 (0, 0.1)	115 (108, 122)	116 (109, 123)	Q1 (0, 0.1)	110 (103, 117)	111 (105, 118)
Q2 (0.1, 0.2)	114 (107, 121)	114 (107, 121)	Q2 (0.1, 0.1)	112 (106, 119)	111 (105, 118)
Q3 (0.2, 0.4)	114 (107, 121)	114 (107, 121)	Q3 (0.1, 0.2)	115 (109, 123)	115 (109, 122)
Q4 (0.4, 19.2)	114 (107, 121)	113 (107, 120)	Q4 (0.3, 8.8)	116 (109, 123)	116 (109, 123)
P-trend^2^	0.87	0.68	P-trend^2^	0.21	0.25

Abbreviations: *MEP*, monoethyl phthalate; *MBP*, mono-n-butyl phthalate; *MiBP*, mono-isobutyl phthalate; *MBzP*, monobenzyl phthalate; *MCPP*, mono-3(carboxypropyl) phthalate; *MCOP*, monocarboxyisooctyl phthalate; *MCNP*, monocarboxyisononyl phthalate; *DEHP*, di-2(ethylhexyl) phthalate

^1^Adjusted models control for maternal age (years), overweight/obese (yes, no), total physical activity (hr/week), race (white, non-white), family history of diabetes (yes, no), infertility diagnosis (male factor, female factor, unexplained) and number of fetus (1, 2)

^2^Tests for linear trend were performed using the log SG-adjusted concentrations in each quartile as a continuous variable in the model.

^3^Sixteen missing urine values for MCOP and MCNP in the 1st trimester, 19 missing values for MCOP and MCNP in the 2nd trimester.

To assess the robustness of the findings, we conducted several sensitivity analyses. First, we excluded 14 women who had 2nd trimester urine samples collected on the same day as the GCT, as well as 85 women without both 1st and 2nd trimester urine samples. In this remaining subset of 159 women, we found similar trimester-specific associations for MEP and MiBP and glucose (See Additional file 1: Table S1). Second, we conducted a sensitivity analysis evaluating the associations between urinary phthalate metabolite concentrations averaged across the 1st and 2nd trimesters in pregnancy and glucose levels from the GCT in the same subset of 159 women. Again, similar associations were seen, with the addition of an inverse association seen for MCOP and glucose levels (See Additional file 1: Table S1). Third, additional adjustments for dietary factors did little to impact these associations (Additional file 1: Table S2). Fourth, we evaluated associations between urinary phthalate metabolite concentrations and an indicator of impaired glucose tolerance (categorized GCT glucose ≥ 140 mg/dL v. < 140 mg/dL). For this, we found a suggestion of 2nd trimester concentrations of MEP and MiBP being associated with an increased and decreased odds of impaired glucose tolerance, respectively (adj. OR for 2nd trimester MEP: 2.48; 95% CI: 0.86–7.20 and adj. OR for 2nd trimester MiBP: 0.37; 95% CI: 0.12, 1.16). Fifth, similar associations were also seen in a restricted analysis of singleton-only births (mean glucose levels for Q4 for MEP: 120 mg/dL; 95% CI: 113, 117 versus Q1: 109 mg/dL; 95% CI: 102, 115). Sixth, adjustment for year of urine sample collection also yielded similar associations, as did additional analyses that excluded women taking metformin at baseline (*n* = 8) and women with physician-diagnosed PCOS (*n* = 21) (data not shown).

## Discussion

In this study of women from a fertility clinic, we found early 2nd trimester MEP concentrations to be positively associated with glucose levels during the 2nd trimester. Women with the highest concentrations of MEP had average glucose levels 12 mg/dL higher than women with the lowest concentrations of MEP. On the other hand, concentrations of MiBP were inversely associated with glucose levels, with average glucose levels 14 mg/dL lower in women with the highest concentrations of MiBP. Younger, potentially more fertile, women (< 37 years) with higher MiBP concentrations had significantly lower glucose levels. The associations between MEP or MiBP and glucose levels remained, despite additional adjustment for dietary factors and sensitivity analyses. Similar associations were seen even when urinary phthalate metabolites were averaged across the two trimesters. With the parent compounds of MEP and MiBP being present in numerous consumer products, exposure to these parent compounds could potentially increase the risk of glucose dysregulation for women seeking treatment for infertility.

Few epidemiological studies have evaluated the associations between urinary phthalate metabolites and glucose levels in pregnant women. One study did not find associations of first trimester phthalate metabolite concentrations with GDM or glucose intolerance [31]. These findings are similar to ours, in that we also did not observe associations between any of the first trimester urinary phthalate metabolite concentrations and 2nd trimester glucose levels. However, our findings differ from the Shapiro et al. study, as we found 2nd trimester concentrations of MEP to be positively associated with glucose levels and 2nd trimester MiBP levels to be inversely associated with glucose levels. On the other hand, another study evaluated this question looking at 2nd trimester concentrations and GDM and found no association, but the analysis may have been underpowered with only 72 women in the study [32]. Additional work is needed to understand how timing of exposure could impact adverse pregnancy outcomes. This is particularly true with GDM and glucose levels during pregnancy, where the 1st trimester is a more insulin-sensitive time period while the 2nd trimester is a more insulin-resistant time period [43].

Several epidemiological studies of non-pregnant populations have found associations between higher phthalate metabolite concentrations and elevated glucose levels and insulin resistance [12, 14, 44]. For example, in a cross-sectional study of men and women participating in the U.S. National Health and Nutrition Examination Survey, higher concentrations of MiBP, MCPP, and ∑DEHP were associated with higher glucose levels, and MEP, MCPP, MnBP, MiBP, MCPP and ∑DEHP were associated with increased insulin resistance, as measured by the homeostatic model assessment of insulin resistance (HOMA-IR) [12]. In a separate study by Dirinck et al., higher concentrations of MEP and MCPP were associated with increased HOMA-IR and Belfiore indices, both indicators of insulin resistance [44]. However, some studies have found no association between phthalates and diabetes-related factors [45]. As such, future work would provide useful insight to better understand whether these chemicals have an impact on glucose regulation, particularly in pregnancy.

MEP may work to increase insulin resistance through its posited estrogenic activity [46]. Urinary concentrations of another EDC with estrogenic activity, bisphenol A, have been shown to be positively associated with glucose levels in human populations [11]. Women seeking treatment for infertility with underlying PCOS, a population with increased insulin resistance, could be particularly affected by exposure to EDCs that alter glucose metabolism [47]. In fact, 18% of this population had impaired glucose tolerance defined in this study as glucose levels ≥ 140 mg/dL based on the 50-g GCT, compared to more average-risk populations, with approximately 5–10% impaired glucose tolerance [31, 33].

On the other hand, the inverse association between MiBP and glucose levels was unexpected, as previous studies have suggested a positive association between MiBP and diabetes and its risk factors [12, 13]. However, MiBP is associated with fish consumption [48], which could be indicative of a healthier diet for women with elevated levels of MiBP. As such, higher MiBP could be associated with reduced risk of GDM due to healthier dietary patterns [49]. While we controlled for dietary patterns as a secondary analysis—with little effect on these findings—it is possible that residual confounding could remain an issue. We saw effect modification by maternal age based on rapid decline in fertility status (< 37 years v. ≥ 37 years). While this finding could be due to chance; it is also possible that healthier lifestyle, as well as the agonistic properties of peroxisome proliferator-activated receptor gamma could be involved in improving insulin sensitivity in women with greater fertility or those with healthier lifestyle. That said reasons for this association are unknown and should be cautiously interpreted, as this requires further assessment in future studies. [50].

The present study has several strengths. First, we used a prospective study design to evaluate the associations between urinary phthalate metabolites and glucose levels during pregnancy, which minimized the possibility of reverse causation. To ensure this, we conducted a sensitivity analysis excluding women who had same-day urine samples in 2nd trimester, finding similar associations. Second, we evaluated pregnancy phthalate metabolite concentrations at two different time points as predictors of later second trimester glucose levels from a standard glucose challenge tests. Third, we assessed these associations in a higher-risk population, women seeking infertility treatment. Fourth, we were able to adjust for a number of potential confounders, as well as assess effect modification, finding age to be an effect modifier of the association between MiBP and lower glucose levels.

Despite these strengths, this study has a number of limitations. First, we evaluated urinary phthalate metabolites in spot urine samples collected at a single time point in the first and second trimesters. Phthalates are non-persistent chemicals and the urinary concentrations of their biomarkers are known to have low to moderate variability [51, 52]; as such, exposure misclassification may exist. Like other pregnancy cohorts studies [51, 53], the correlation between phthalate metabolite concentrations in the first and second trimesters was moderate in the present study. That said, we evaluated multiple time periods finding differing associations, which may be suggestive of sensitive windows of exposure with respect to the association between phthalates and glucose levels in pregnancy. While we hypothesize that second trimester may be a sensitive time period, to account for variability in phthalate exposure across pregnancy and the robustness of these findings, we assessed urinary phthalate metabolite concentrations, averaged across 1st and 2nd trimesters, and glucose levels. We found similar associations for both average and trimester-specific urinary phthalate metabolite concentrations and pregnancy glucose levels. Furthermore, we conducted a number of sensitivity analyses, including additional adjustments for diet, year of phthalate analysis, as well as restricted analyses for excluding women with same-day urine sample collection, metformin use, and PCOS. We found similar associations in these sensitivity analyses. Second, we did not adjust for pregnancy weight gain in this analysis. Gestational weight gain is likely a potential mediator, by which adjustment would lead to inaccurate findings. However, one previous study did not find an association between urinary phthalate metabolites and weight gain in pregnancy. Third, we did not evaluate the clinical diagnosis of GDM in this analysis due to being underpowered, with only 10 women being diagnosed with this condition. However, we assessed impaired glucose tolerance and found higher concentrations of MEP to be associated with an increased odds of impaired glucose tolerance defined as having a GCT glucose level > 140 mg/dL. Fourth, we evaluated this question in a high-risk population—women treated at a fertility clinic. As such, our findings may not be generalizable to women without fertility issues. However, assessing a potentially modifiable risk factor of higher glucose levels in pregnancy in a population with a higher baseline risk of impaired glucose tolerance and GDM could provide helpful information to reduce risk of elevated glucose levels and its sequelae.

## Conclusion

In conclusion, in this study, we found some evidence that MEP measured in the 2nd trimester was associated with higher glucose levels on routine obstetric testing. MEP, a metabolite of the prevalent diethyl phthalate commonly used in personal care products and a marker of certain lifestyle factors, was associated with elevated glucose levels. Conversely, MiBP, a metabolite of diisobutyl phthalate (found in food and consumer products such as fish, raincoats, nail polish, and food wrap) was associated with decreased glucose levels. While further research is needed, our results suggest that phthalate exposure may be a potentially modifiable risk factor of glucose dysregulation among women seeking treatment for infertility.

## Additional files

Additional file 1: **Table S1.** Association between urinary phthalate metabolites and glucose levels in pregnancy from 24 to 28 week GCT among women with prospectively-collected urine samples from 1st and 2nd trimester (*n* = 159). Legend: Abbreviations: MEP, monoethyl phthalate; MBP, mono-n-butyl phthalate; MiBP, mono-isobutyl phthalate; MBzP, monobenzyl phthalate; MCPP, mono(3-carboypropyl) phthalate; MCOP, monocarboxyisooctyl phthalate; MCNP, monocarboxyisononyl phthalate; DEHP, di(2-ethylhexyl) phthalate. ^1^Models were adjusted for maternal age (years), overweight/obese (yes, no), total physical activity (hr/week), race (white, non-white), family history of diabetes (yes, no), infertility diagnosis (male factor, female factor, unexplained) and number of fetus (1,2). ^2^Tests for linear trend were performed using the log SG-adjusted concentrations in each quartile as a continuous variable in the model. **Table S2.** Restricted analysis with additional adjustment for diet for associations between mean glucose and MEP and MiBP for dietary patterns. Legend: Abbreviations: MEP, monoethyl phthalate; MBP, mono-n-butyl phthalate; MiBP, mono-isobutyl phthalate; MBzP, monobenzyl phthalate; MCPP, mono(3-carboypropyl) phthalate; MCOP, monocarboxyisooctyl phthalate; MCNP, monocarboxyisononyl phthalate; DEHP, di(2-ethylhexyl) phthalate. ^1^Models were adjusted for maternal age (years), overweight/obese (yes, no), total physical activity (hr/week), race (white, non-white), family history of diabetes (yes, no), infertility diagnosis (male factor, female factor, unexplained) and number of fetus (1,2). ^2^Additionally adjusted for Prudent and Western dietary patterns (DOCX 27 kb).

## Abbreviations

DEHP: di (2-ethylhexyl) phthalate
EARTH: Environment and Reproductive Health Study
EDC: Endocrine-disrupting chemicals
GDM: Gestational Diabetes Mellitus
MBP: Mono-n-butyl phthalate
MBzP: Monobenzyl phthalate
MCNP: Monocarboxyisononyl phthalate (MCNP)
MCOP: Monocarboxyisooctyl phthalate
MCPP: Mono (3-carboypropyl) phthalate
MECPP: Mono (2-ethyl-5-carboxypentyl) phthalate
MEHHP: Mono (2-ethyl-5-hydroxyhexyl) phthalate
MEHP: Mono (2-ethylhexyl) phthalate
MEOHP: Mono (2-ethyl-5-oxohexyl) phthalate
MEP: Monoethyl phthalate
MiBP: Mono-isobutyl phthalate
QA/QC: Quality assurance/quality control
SG: Specific gravity
