# Supplementary material for: Trimester-specific phthalate concentrations and glucose levels among women from a fertility clinic
Source: Environ Health. 2018 Jun 14;17:55. doi: 10.1186/s12940-018-0399-5 (PMC6000948; doi:10.1186/s12940-018-0399-5)
Supplement: Supplementary file 1 — Table S1. Association between urinary phthalate metabolites and glucose levels in pregnancy from 24 to 28 week GCT among women with prospectively-collected urine samples from 1st and 2nd trimester (n = 159). Legend: Abbreviations: MEP, monoethyl phthalate; MBP, mono-n-butyl phthalate; MiBP, mono-isobutyl phthalate; MBzP, monobenzyl phthalate; MCPP, mono(3-carboypropyl) phthalate; MCOP, monocarboxyisooctyl phthalate; MCNP, monocarboxyisononyl phthalate; DEHP, di(2-ethylhexyl) phthalate. 1Models were adjusted for maternal age (years), overweight/obese (yes, no), total physical activity (hr/week), race (white, non-white), family history of diabetes (yes, no), infertility diagnosis (male factor, female factor, unexplained) and number of fetus (1,2). 2Tests for linear trend were performed using the log SG-adjusted concentrations in each quartile as a continuous variable in the model. Table S2. Restricted analysis with additional adjustment for diet for associations between mean glucose and MEP and MiBP for dietary patterns. Legend: Abbreviations: MEP, monoethyl phthalate; MBP, mono-n-butyl phthalate; MiBP, mono-isobutyl phthalate; MBzP, monobenzyl phthalate; MCPP, mono(3-carboypropyl) phthalate; MCOP, monocarboxyisooctyl phthalate; MCNP, monocarboxyisononyl phthalate; DEHP, di(2-ethylhexyl) phthalate. 1Models were adjusted for maternal age (years), overweight/obese (yes, no), total physical activity (hr/week), race (white, non-white), family history of diabetes (yes, no), infertility diagnosis (male factor, female factor, unexplained) and number of fetus (1,2). 2Additionally adjusted for Prudent and Western dietary patterns (DOCX 27 kb). [file 12940_2018_399_MOESM1_ESM.docx]

**Table S1 Association between urinary phthalate metabolites and glucose levels in pregnancy from 24-28 week GCT among women with prospectively-collected urine samples from 1^st^ and 2^nd^ trimester (n=159)**

|  | Population means of blood glucose in mg/dL (95%CI) | | | | | |
| --- | --- | --- | --- | --- | --- | --- |
| Geometric mean of SG-adjusted phthalate metabolites (range) | Average across 1^st^ and 2^nd^ trimesters | | 1^st^ trimester | | 2^nd^ trimester | |
|  | Unadjusted | Adjusted ^1^ | Unadjusted | Adjusted ^1^ | Unadjusted | Adjusted ^1^ |
| MEP (µg/L) |  |  |  |  |  |  |
| Q1 (3.6, 16.3) | 107 (100, 114) | 109 (102, 117) | 107 (100, 115) | 110 (103, 117) | 109 (102, 116) | 109 (102, 116) |
| Q2 (16.5, 34.2) | 118 (111, 127) | 118 (111, 126) | 120 (113, 129) | 122 (115, 130) | 109 (103, 117) | 111 (104, 119) |
| Q3 (34.3, 103) | 112 (105, 120) | 114 (107, 121) | 110 (103, 118) | 109 (103, 117) | 116 (108, 124) | 116 (109, 123) |
| Q4 (104, 13500) | 123 (115, 131)* | 119 (111, 127) | 120 (112, 128) | 117 (109, 124) | 126 (118, 135)* | 124 (116, 132)* |
| P-trend^2^ | 0.02 | 0.17 | 0.09 | 0.57 | 0.0004 | 0.004 |
| MBP (µg/L) |  |  |  |  |  |  |
| Q1 (<LOD, 7.1) | 112 (104, 120) | 112 (105, 119) | 114 (106, 122) | 116 (108, 124) | 112 (105, 120) | 111 (104, 119) |
| Q2 (7.1, 11.2) | 118 (110, 126) | 117 (109, 125) | 114 (106, 122) | 112 (104, 119) | 113 (106, 121) | 114 (106, 121) |
| Q3 (11.2, 17.3) | 113 (106, 121) | 115 (107, 122) | 118 (110, 126) | 117 (110, 125) | 115 (108, 124) | 116 (109, 124) |
| Q4 (17.4, 2342) | 116 (109, 125) | 117 (109, 124) | 113 (105, 121) | 113 (106, 121) | 119 (112, 128) | 118 (111, 126) |
| P-trend^2^ | 0.56 | 0.45 | 0.94 | 0.86 | 0.19 | 0.18 |
| MiBP (µg/L) |  |  |  |  |  |  |
| Q1 (<LOD, 3.7) | 117 (109, 125) | 118 (110, 126) | 122 (114, 130) | 122 (114, 130) | 121 (113, 130) | 122 (114, 130) |
| Q2 (3.7, 6.2) | 116 (108, 124) | 117 (110, 125) | 111 (104, 119) | 113 (106, 120) | 113 (105, 121) | 113 (106, 121) |
| Q3 (6.2, 10.6) | 119 (111, 127) | 119 (112, 127) | 114 (106, 122) | 113 (106, 121) | 117 (110, 126) | 119 (112, 127) |
| Q4 (10.7, 56.6) | 108 (101, 116) | 106 (99, 113)* | 112 (105, 120) | 110 (103, 118) | 109 (102, 116)* | 107 (100, 114)* |
| P-trend^2^ | 0.16 | 0.04 | 0.17 | 0.05 | 0.05 | 0.01 |
| MBzP (µg/L) |  |  |  |  |  |  |
| Q1 (<LOD, 1.6) | 114 (107, 122) | 114 (107, 122) | 112 (104, 120) | 111 (104, 119) | 110 (103, 118) | 110 (103, 118) |
| Q2 (1.6, 3.1) | 109 (102, 117) | 108 (102, 115) | 118 (110, 126) | 119 (111, 127) | 111 (104, 119) | 112 (105, 120) |
| Q3 (3.1, 5.4) | 114 (107, 122) | 116 (109, 124) | 111 (103, 118) | 110 (103, 118) | 118 (110, 126) | 117 (110, 125) |
| Q4 (5.4, 305) | 123 (115, 131) | 122 (114, 130) | 118 (110, 126) | 117 (110, 125) | 121 (113, 129)* | 120 (112, 128) |
| P-trend^2^ | 0.07 | 0.08 | 0.47 | 0.52 | 0.04 | 0.05 |
| MCPP (µg/L) |  |  |  |  |  |  |
| Q1 (<LOD, 1.8) | 113 (106, 121) | 113 (106, 121) | 116 (108, 124) | 117 (109, 124) | 111 (104, 119) | 113 (105, 120) |
| Q2 (1.8, 4) | 124 (116, 132) | 125 (117, 133) | 116 (108, 124) | 116 (109, 124) | 121 (113, 130) | 120 (112, 128) |
| Q3 (4.2, 13.2) | 113 (106, 121) | 113 (106, 120) | 119 (111, 127) | 118 (111, 126) | 113 (106, 121) | 114 (107, 121) |
| Q4 (13.7, 698) | 110 (103, 118) | 109 (103, 116) | 108 (100, 115) | 107 (100, 114) | 114 (107, 122) | 114 (107, 122) |
| P-trend^2^ | 0.25 | 0.09 | 0.16 | 0.07 | 0.93 | 0.9 |
| MCOP (µg/L)^3^ |  |  |  |  |  |  |
| Q1 (<LOD, 11.1) | 117 (109, 126) | 116 (109, 125) | 116 (108, 125) | 115 (108, 124) | 118 (110, 127) | 119 (111, 128) |
| Q2 (11.3, 29.8) | 118 (109, 126) | 120 (112, 129) | 117 (109, 125) | 117 (109, 125) | 107 (99, 115) | 108 (101, 115) |
| Q3 (30.7, 79.8) | 114 (106, 123) | 112 (105, 120) | 117 (109, 125) | 116 (109, 124) | 117 (109, 126) | 117 (109, 125) |
| Q4 (83.9, 736) | 107 (100, 115) | 107 (100, 115) | 104 (97, 112) | 105 (98, 113) | 114 (107, 123) | 113 (106, 121) |
| P-trend^2^ | 0.07 | 0.04 | 0.05 | 0.06 | 0.95 | 0.65 |
| MCNP (µg/L)^3^ |  |  |  |  |  |  |
| Q1 (<LOD, 2) | 113 (105, 121) | 114 (106, 122) | 113 (105, 121) | 113 (105, 121) | 114 (106, 122) | 116 (108, 125) |
| Q2 (2, 3.6) | 124 (116, 133) | 124 (116, 133) | 118 (110, 127) | 118 (110, 126) | 116 (108, 125) | 113 (106, 121) |
| Q3 (3.6, 8.2) | 106 (99, 114) | 105 (98, 112) | 111 (104, 120) | 110 (103, 118) | 113 (105, 121) | 115 (108, 124) |
| Q4 (8.2, 256) | 113 (106, 122) | 114 (106, 122) | 111 (104, 120) | 113 (105, 121) | 114 (106, 122) | 112 (104, 119) |
| P-trend^2^ | 0.48 | 0.36 | 0.57 | 0.65 | 0.86 | 0.51 |
| ∑DEHP |  |  |  |  |  |  |
| Q1 (0, 0.1) | 112 (105, 120) | 112 (105, 119) | 117 (110, 126) | 118 (111, 126) | 110 (103, 118) | 111 (104, 119) |
| Q2 (0.1, 0.2) | 116 (109, 125) | 116 (109, 124) | 113 (106, 121) | 113 (106, 121) | 116 (109, 124) | 115 (108, 123) |
| Q3 (0.2, 0.4) | 115 (107, 123) | 114 (107, 122) | 115 (107, 123) | 114 (107, 122) | 116 (108, 124) | 115 (107, 122) |
| Q4 (0.4, 19.2) | 116 (109, 125) | 118 (110, 126) | 112 (105, 120) | 112 (105, 120) | 117 (110, 126) | 119 (112, 127) |
| P-trend^2^ | 0.52 | 0.35 | 0.45 | 0.41 | 0.28 | 0.17 |

^1^Adjusted models control for maternal age (continuous), body mass index (continuous), smoking status (never, ever), race (white, non-white), education levels (some college or technical school or less, college graduate or higher), infertility diagnosis (male factor, female factor, unexplained) and number of fetus (1, 2)

^2^Tests for linear trend were performed using the log SG-adjusted concentrations in each quartile as a continuous variable in the model.

**Table S2 Restricted analysis with additional adjustment for diet for associations between mean glucose and MEP and MiBP for dietary patterns.**

| **SG-adjusted phthalate metabolites** | **Population means of blood glucose in mg/dL (95%CI)** | | | |
| --- | --- | --- | --- | --- |
|  | Concentration measured at the 1^st^ trimester | | Concentration measured at the 2^nd^ trimester | |
|  | Adjusted^1^(n=164) | Adjusted^2^ (n=164), additional adjusted for dietary patterns | Adjusted^1^ (n=160) | Adjusted^2^ (n=160), additional adjusted for dietary patterns |
| **MEP (µg/L)** |  |  |  |  |
| Q1 | 116 (108, 125) | 116 (108, 125) | 110 (103, 119) | 110 (103, 118) |
| Q2 | 113 (106, 121) | 113 (106, 121) | 111 (103, 119) | 110 (103, 118) |
| Q3 | 106 (99, 113) | 106 (100, 113) | 110 (103, 118) | 111 (104, 118) |
| Q4 | 120 (112, 128) | 119 (112, 127) | 123 (115, 131) | 123 (115, 131) |
| P-trend^2^ |  |  | 0.03 | 0.02 |
| **MiBP (µg/L)** |  |  |  |  |
| Q1 | 114 (106, 123) | 114 (107, 123) | 119 (110, 128) | 118 (110, 127) |
| Q2 | 115 (107, 124) | 115 (108, 124) | 112 (105, 120) | 112 (104, 120) |
| Q3 | 116 (109, 124) | 117 (109, 125) | 119 (112, 127) | 120 (112, 129) |
| Q4 | 109 (102, 116) | 109 (102, 116) | 106 (99, 113) | 106 (99, 113) |
| P-trend^2^ |  |  | 0.07 | 0.08 |

^1^Adjusted models control for maternal age (continuous), body mass index (continuous), smoking status (never, ever), race (white, non-white), education levels (some college or technical school or less, college graduate or higher), infertility diagnosis (male factor, female factor, unexplained) and number of fetus (1, 2)

^2^Additionally adjusted for Prudent and Western dietary patterns
